# A Signal Processing Algorithm of Two-Phase Staggered PRI and Slow Time Signal Integration for MTI Triangular FMCW Multi-Target Tracking Radars

**DOI:** 10.3390/s21072296

**Published:** 2021-03-25

**Authors:** Taiwen Tang, Chen Wu, Janaka Elangage

**Affiliations:** Defence Research and Development Canada, Ottawa Research Centre, Ottawa, ON K1A 0Z4, Canada; chen.wu@forces.gc.ca (C.W.); janaka.elangage@forces.gc.ca (J.E.)

**Keywords:** staggered PRI, slow time integration, FMCW, digital beamforming, radar electronic warfare

## Abstract

In this paper, a novel signal processing algorithm for mitigating the radar blind speed problem of moving target indication (MTI) for frequency modulated continuous wave (FMCW) multi-target tracking radars is proposed. A two-phase staggered pulse repetition interval (PRI) solution is introduced to the FMCW radar system. It is implemented as a time-varying MTI filter using twice the hardware resources as compared to a uniform PRI MTI filter. The two-phase staggered PRI FMCW waveform is still periodic with a little more than twice the period of the uniform PRI radar. We also propose a slow time signal integration scheme for the radar detector using the post-fast Fourier transformation Doppler tracking loop. This scheme introduces 4.77 dB of extra signal processing gain to the signal before the radar detector compared with the original uniform PRI FMCW radar. The validation of the algorithm is done on the field programmable logic array in the loop test bed, which accurately models and emulates the target movement, line of sight propagation and radar signal processing. A simulation run of tracking 16 s of the target movement near or at the radar blind speed shows that the total degradation from the raw post-fast Fourier transformation received signal to noise ratio is about 2 dB. With a 20 dB post-processing signal to noise ratio of the proposed algorithm for the moving target at around a 20 km range and with about a −3.5 dB m^2^ radar cross section at a 1.5 GHz carrier frequency, the tracking errors of the two-dimensional angles with a 4×4 digital phased array are less than 0.2 degree. The range tracking error is about 28 m.

## 1. Introduction

Advanced radar sensors that feature multiple functions, multiple modes, multiple channels, multiple waveforms and multiple targets have been researched in recent decades [1,2,3]. These advanced radars are also equipped with “smart” processors that adapt to the sensing environments and sensing requirements. In recent years, this technology trend of advanced radars has been realized primarily because of the combination of advanced signal processing, advanced radio frequency (RF) and integrated circuit technology and artificial intelligence. In this paper, we focus on some advanced signal processing techniques.

Frequency modulated continuous wave (FMCW) radar has been widely used for both defense and civilian applications [1,4,5]. In this paper, we focus on target tracking radars that can continuously track the movement of the intended targets. The final goal of our research is to build a ground-based experimental FMCW phased array tracking radar that can track fast-moving aerial targets at a 10~40 km range and use it for radar and electronic warfare technique studies. The radar is designed for L band (1–2 GHz) with a narrow bandwidth and potentially frequency hopping capability if the radio frequency front-end permits.

For FMCW, the radar frequency sweeps with time. Particularly, linear FMCW uses linear frequency sweeping slopes. Well-known linear FMCW waveforms include sawtooth FMCW and triangular FMCW. Using a triangular FMCW waveform is the focus of this paper. The advantages of the triangular FMCW waveform lie in three aspects. First, the peak-to-average power ratio of the waveform is much lower than the time domain modulated pulse waveforms. Second, the continuous transmission provides high signal processing gain that can be leveraged by fast Fourier transformation (FFT). Thirdly, it includes upbeat sweeps and downbeat sweeps, which makes it easier to estimate the range and Doppler values at the same time as the sawtooth waveform. One of the design challenges for the FMCW radar is coping with power leakage from the transmitter (TX) to receiver (RX). Another challenge is how to combine FMCW with the phased array technology.

Frequency modulation of the transmitted waveform can be done in the digital domain or in the analog domain. Traditionally, automotive radars and harbor surveillance radars use analog frequency modulation in the transmitter [6,7,8,9,10,11,12,13,14,15,16,17]. Digital frequency modulation at the transmitter is a new trend that provides better signal fidelity than analog frequency modulation, because in digital frequency modulation there is almost no sweep nonlinearity that can be introduced by the analog domain modulation. Sweep nonlinearity degrades the range resolution of FMCW radar. Analysis of sweep nonlinearity has been seen in [18]. In this paper, we focus on digital frequency modulation.

Phased array technology has been used in modern radars for many decades. From passive electronically scanned array (PESA) to active electronically scanned array (AESA), the radar transmitter employs much higher total transmit power and can detect targets at a much longer range using AESA than using PESA [19]. Digital phased array (DPA) is an advanced receiver technique. This scheme uses a per-antenna analog-to-digital converter (ADC). With DPA, modern radars can perform multiple functions, e.g., search function and track function, and track multiple targets [5]. In this paper, our focus is AESA with DPA techniques.

At the receiver of the radar with DPA, four-dimensional (4D) FFT processing can be done to estimate the range, the two-dimensional (2D) angles and the Doppler of the targets [12,20]. The 4D FFT processing is an open loop processing technique. After the 4D FFT, the system requires a track formulation module to form the tracking trajectories of different targets [21,22,23]. In some cases, the open loop tracking radar system performs the dual function of both searching and tracking. The closed loop tracking schemes, which are seen in [24,25,26,27], require initial input parameters, such as the initial range, the initial 2D angles and the initial Doppler of an intended target, to establish a track. After establishing the track, the closed loop tracking radar relies on its signal processing to track the target. In [27], an FMCW target-tracking radar is proposed and closed loop tracking of the target range and angles is applied to this system. This FMCW radar uses a constant false alarm rate (CFAR) detector [28,29] and 2D monopulse angle tracking [30,31].

To combat the strong stationary clutter signals, a moving target indication (MTI) filter is used to cancel the clutter signal [25,32,33,34,35]. When the system uses a uniform pulse repetition interval (PRI), the system experiences significant signal to noise ratio (SNR) drops in the radar blind speed zone [25]. In the FMCW context, PRI also refers to the period of the waveform. In [27], we also observe the blind speed phenomenon with the triangular FMCW waveform and MTI.

To solve the radar blind speed issue, radar PRI staggering [36,37] and carrier frequency hopping are both effective. In this paper, we focus on the staggered PRI solution which has more than one timing phase in each PRI. Here, timing phases, or simply phases, mean the time intervals of different durations of waveform segments but not signal phases. In the context of pulse-Doppler radar, it is straightforward to implement the staggered PRI scheme. The radar simply transmits a short pulse followed by a variable-duration silence time in each PRI. For FMCW radars, implementation of staggered PRI transmission can have many different methods [38,39,40]. On the other hand, since staggered PRI schemes still suffer certain SNR drops in the radar blind speed zone, extra processing gain from slow time signal integration, e.g., [41], is desirable. However, implementing many staggering phases for the staggered PRI schemes makes the radar signal period long. With a long radar signal period, high processing gain from slow time signal integration is not easy to achieve. This is because we need to implement a fine Doppler grid for FFT processing in the Doppler domain, and have clean angle estimation results, because angle estimation errors can degrade the quality of the FFT processing in the Doppler domain. In this paper, we propose a two-phase staggered (TPS) PRI MTI (TPS-PRI-MTI) scheme that is different from the prior art. The two phases are two different timing intervals of two consecutive FMCW periods. The other main novelty of this paper is that we combine the staggered PRI scheme and the slow time signal integration scheme, which can achieve an extra processing gain of 4.77 dB in the blind speed region in the Doppler domain. The proposed algorithm improves the tracking performance of the algorithm in [27] in the radar blind speed zone. It can be seamlessly integrated with the range extrapolation method proposed in [27] to further improve the target tracking performance.

This paper is organized as follows. In Section 2, the FMCW radar transmitter is presented and the novel TPS triangular FMCW waveform is proposed. Section 3 presents the receiver system processing architecture and algorithms. In Section 4, the field programmable gate array (FPGA)-based simulation test bed that can speed up computer-based simulation is introduced, the simulation results are presented and some discussion about the results is also given. Finally, in Section 5, some conclusions about our design are presented.

## 2. The FMCW Radar Transmitter

In this section, initially, the FMCW radar system overview is given in Section 2.1. Then the AESA transmitter design is discussed in Section 2.2. After that, the chirp function used to form the FMCW waveform is introduced in Section 2.3. The novel TPS triangular FMCW waveform is proposed in Section 2.3. Lastly, the digital modulation technique for generating the TPS triangular FMCW waveform is discussed in Section 2.4.

### 2.1. The FMCW Radar System Overview

**Figure 1:**
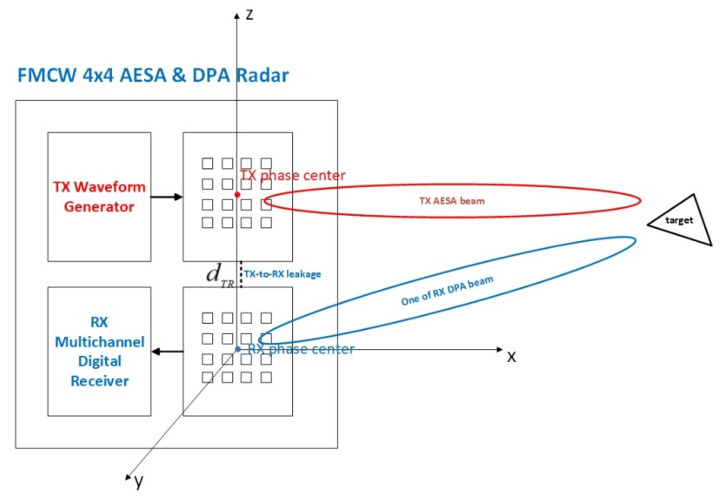
The active electronically scanned array (AESA) transmitter and the digital phased array (DPA) receiver with separate transmitter (TX) and receiver (RX) antenna panels. Both the TX and RX antenna panels have four-by-four element phase arrays. The RX and TX antenna phase centers are plotted as blue and red dots, respectively. The distance between the TX and the RX phase centers is$\;d_{TR}$. The origin of the radar coordinate system is located at the RX phase center.

The high-level FMCW radar system diagram is shown in Figure 1. The TX is an AESA. Each TX element is connected to its own power amplifier, an up-conversion RF chain and a digital-to-analog converter (DAC). The RX has a DPA. Each RX array element feeds the received signal into a low-noise amplifier (LNA) followed by a down-conversion RF chain and an analog-to-digital converter (ADC). The TX and the RX have separate antenna panels, which give sufficient TX and RX separation distance to avoid saturation of the LNAs of all RX chains. The calibration of the gain and phase imbalance of the AESA and DPA is important, but outside the scope of this paper. We just assume perfect gain and phase calibration of the AESA and DPA.

When modeling the radar system and the line-of-sight propagation effects, we model the “virtual RF” (this term is just used to contrast the baseband of the radar) signals at the TX and the RX in the oversampling domain using the same approach as in [27]. The oversampling factor is two. The TX RF chain and the RX RF chain are abstracted as low-pass filters. We represent both the TX RF chain and the RX RF chain by finite impulse response (FIR) half-band filters [42]. The orders of the half-band filters are shown in Section 4.2. We also model the receiver noise as additive white Gaussian noise (AWGN). The noise power density with a noise figure is also shown in Section 4.2.

The TX-to-RX leakage problem in the FMCW radar system has been addressed in [27]. The solution is to use bi-static radar instead of mono-static radar for the FMCW waveform. The TX and the RX antennas are separated by certain distances to guarantee that each LNA for each receive antenna is not saturated. Then, after analog-to-digital conversion, we filter out the direct current (DC) in the digital domain for each RX chain of each RX antenna. The near-DC spurs caused by amplitude modulation of the TX-to-RX leakage signal can be effectively mitigated by the MTI filter. The design problem of integrating the triangular FMCW waveform with the DPA receiver has also been resolved in [27]. The solution is to use 2D digital monopulse angle tracking after the FFTs. In this paper, the receiver architecture proposed in Section 3.1 includes the solutions to both problems.

### 2.2. The AESA Radar Transmitter

**Figure 2:**
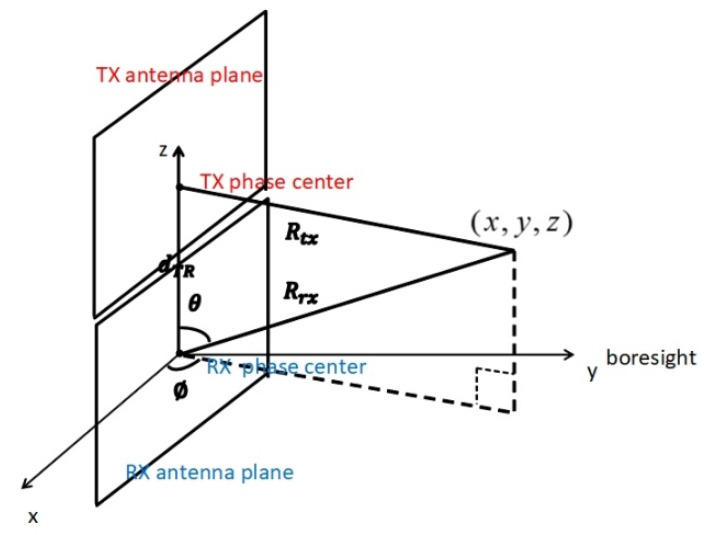
The TX and RX antenna array panels and the target ($R_{rx}$ and $R_{tx}$ are much bigger than $d_{TR}$ in the real system).

The coordinate system in the modeling and simulation of the FMCW tracking and measurement system is presented in Figure 2. The panels of both the TX phased array and the RX phased array are in the XZ-plane. The RX phase reference center is at the origin of the radar coordinate system (the origins of both the Cartesian coordinate and the spherical coordinate). The three-dimensional Cartesian coordinate tuple along the x, y and z axes is denoted by (x,y,z). The corresponding spherical coordinates are denoted by $\left(\phi ,\;\theta ,R_{rx}\right)$, which is also known as azimuth angle (Az) (in radians), elevation angle (El) (in radians) and range ($R_{rx}$) (in meters). The distance between the TX phase center and the RX phase center is denoted by $d_{TR}$. The distance between the TX phase center and the point (x,y,z) is denoted by $R_{tx}$. The angle numbers used for both TX and RX beamforming are defined as in Equation (1).

(1) [a(0),a(1)]=[cos(ϕ)sin(θ), cos(θ)].

The coordinate systems of the TX antenna panel for TX beamforming and the RX panel for RX beamforming have different origins. The TX coordinate system’s origin is located at the TX phase center. The RX coordinate system’s origin is located at the RX phase center. The beamforming process for RX follows the description in Section 2.5 of [27]. The TX beamforming process is the same. However, the TX beamforming angle numbers $a_{tx}\left(0\right)$ and $a_{tx}\left(1\right)$ are defined with respect to the Az and El in the TX spherical coordinates. Since $d_{TR}$ is small compared to $R_{rx}$ and $R_{tx}$, $a_{tx}\left(0\right)\approx a\left(0\right)$ and $a_{tx}\left(1\right)\approx a\left(1\right).$

For a moving target that is of interest in our tracking radar, we feed initial parameters of the moving target to the radar. These initial parameters are estimates of the initial range, the initial angles in azimuth and elevation and the initial normalized Doppler (normalized by the baseband sampling frequency $f_{B}$). They are assumed to be obtained by a target acquisition radar. The estimates do not need to be perfect, however, the estimation errors cause degradation to the post-processing signal to noise ratio (SNR). These initial parameters are denoted by Rinit (initial range parameter), ainit(0) (the initial azimuth angle number parameter), ainit(1) (the initial elevation angle number parameter) and ${\mathrm{Dinit}}_{dop}$ (the initial normalized Doppler parameter).

**Figure 3:**
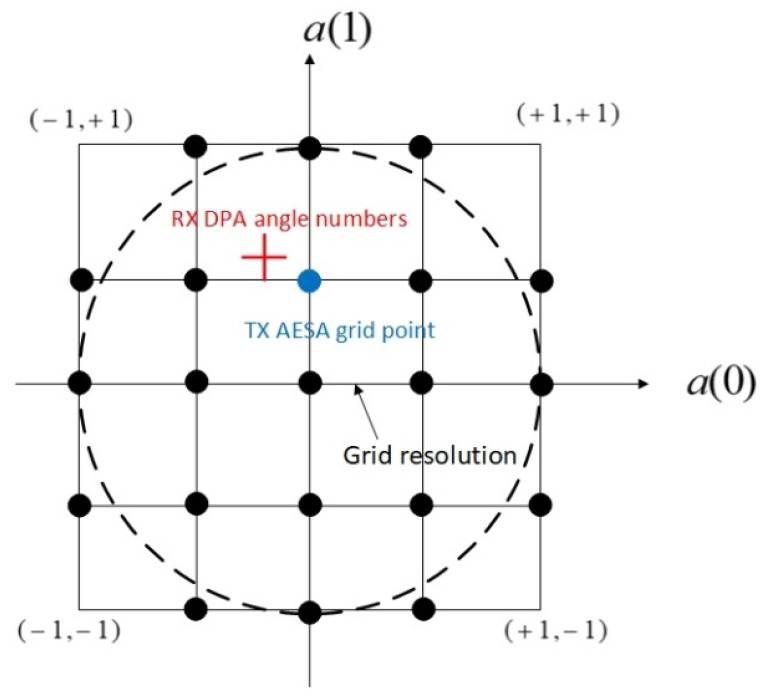
An example of the TX AESA angle number quantization grid. The grid resolution is defined as the distance between two nearest neighbors in the grid. An initial angle number pair or a RX DPA estimated angle number pair for the target is quantized to the nearest grid point. Note that the true target angle numbers satisfy $a{\left(0\right)}^{2}+\;a{\left(1\right)}^{2}<\;1$.

The initial angles are used to set up the initial AESA beamformer in TX and the initial DPA beamformer in RX. For the AESA, these initial angle numbers are quantized to the nearest points in a grid. The TX AESA beamformer uses the angle grid points to form its beamformer. The uniform grid quantizes the initial angle number pair (ainit(0), ainit(1)) to the nearest grid point, as shown in Figure 3. We want to keep the TX beamforming angle numbers constant within one PRI as much as possible to minimize the skin return signal variation for stationary clutters. Since the TX beam follows the target movement, the resolution of the quantization grid needs to be greater than the maximal changes of a(0) and a(1) of the target within one PRI. On the other hand, since the target moves continuously, switching the TX beam from a quantization point in the grid to a nearest neighbor of the point is inevitable. This causes variation of the skin return signal in amplitude and phase for even stationary clutters. This effect is not desirable for the MTI filter. Therefore, we must give an upper bound to the resolution of the grid as well. In our simulations, we set the resolution to $2^{-7}$ for the radar parameters and found that the simulation performance is acceptable. However, this resolution is not the optimized value.

The outputs of the receiver of the tracking radar are range R, angle numbers a(0) and a(1), normalized Doppler $D_{dop}$ and target velocities along the x, y and z axes. Once the tracking is established (normally after a given amount of time on the order of 100 PRIs after feeding the initial parameters if the SNR is sufficient), the AESA uses the angle numbers a(0) and a(1) output by the radar receiver to form its beamformer. This AESA beamforming procedure also utilizes the angle number quantization grid. Using an AESA quantization grid ensures that the AESA does not need to change the TX beamforming angle continuously and benefits the multiple target tracking since a TX beam centered at the grid point may cover multiple targets.

The tracking radar employs multiple target tracking with only one carrier frequency. The receiver architecture will be depicted in Section 3. The AESA can form multiple beams if the quantized a(0)s and a(1)s of different moving targets show different quantization values. In this case, it requires a TX-power-back-off for each beam and reduces the effective isotropic radiation power (EIRP) of each beam. In the worst case, the TX-power-back-off in linear scale is inversely proportional to the square of the number of tracked targets.

### 2.3. TPS Triangular FMCW Waveform

The simple linear FMCW waveform in the baseband has the following mathematical form in the time domain:

(2) $$g_{simple}\left(t\right)=e^{2\pi jbt^{2}}$$

where t  is the time in seconds, b is the chirp rate and $j=\;\sqrt{-1}$. Note that the instantaneous frequency of the chirp function is 2bt, where the frequency sweep slope is 2b. When b is positive, the instantaneous frequency is increasing with time t. This is called upbeat. When b is negative, the instantaneous frequency is decreasing with time t. This is called downbeat.

**Figure 4:**
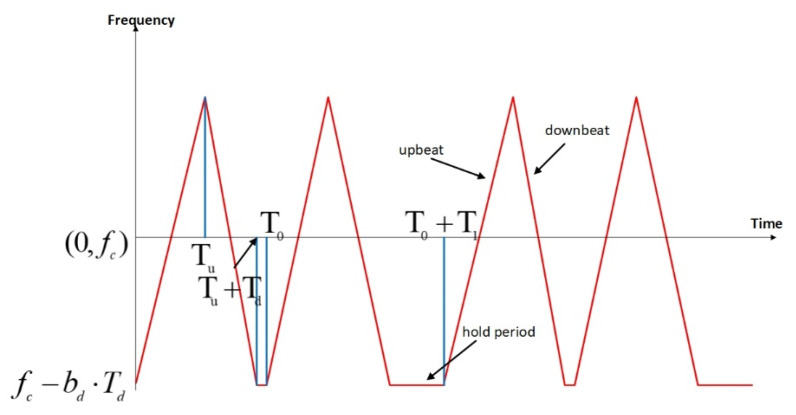
The two-phase staggered (TPS) triangular frequency modulated continuous wave (FMCW) waveform.

The TPS triangular FMCW waveform has an instantaneous frequency f(t) of the following form:

(3) $$f(t)=\left\{\begin{array}{cc} f_{c}+2b_{u}\cdot \left(\mathrm{mod}\left(\mathrm{mod}\left(t,T_{0}+T_{1}\right),T_{u}+T_{d}\right)-0.5T_{u}\right), & \mathrm{for}\mathrm{the}\mathrm{upbeats}\\ f_{c}+2b_{d}\cdot \left(\mathrm{mod}\left(T_{u}+T_{d}-\mathrm{mod}\left(t,T_{0}+T_{1}\right),T_{u}+T_{d}\right)-0.5T_{d}\right), & \mathrm{for}\mathrm{the}\mathrm{downbeats}\\ f_{c}-b_{d}\cdot T_{d}, & \mathrm{for}\mathrm{the}\mathrm{frequency}\mathrm{hold}\mathrm{periods} \end{array}\right.$$

where the notations are summarized in Table 1 and we assume $b_{u}\cdot T_{u}=\;b_{d}\cdot T_{d}$. The frequency of the waveform is also illustrated in Figure 4.

The relation between the instantaneous frequency and the actual waveform is the following

(4) $$g\left(t\right)=e^{2\pi j\int_{0}^{t}f\left(\tau \right)d\tau }$$

The proposed waveform has two time phases, i.e., phase 0 and phase 1. Phase 0 has a duration of $T_{0}$ and consists of a triangular frequency sweep (the upbeat and the downbeat) and a frequency hold period. Note that the upbeat and downbeat can have different chirp rates but the same frequency sweep range. Similarly, phase 1 has a duration of $T_{1}$ and consists of an identical triangular frequency sweep and a frequency hold period with a longer duration than the hold time of phase 0. This waveform is periodic with a period of $T=\;T_{0}+\;T_{1},$ which is the PRI of the FMCW radar. Essentially, it performs non-uniform sampling of the Doppler signature of the moving target [37]. There can be many different implementations of the non-uniform sampling scheme for the FMCW waveform. For example, the hold periods can be replaced by arbitrary functions. The proposed one is simple but very practical for implementation.

### 2.4. Generating TPS Triangular FMCW Waveform Using Digital Modulation

The chirp bandwidth $BW\;=\;2b_{u}T_{u}\;=\;2b_{d}T_{d}$ (BW is the two-sided bandwidth). The baseband sampling rate is $f_{B}=\frac{1}{T_{B}}$, where $T_{B}$ is the baseband sampling interval. Based on the Nyquist sampling theorem (the sampling rate must be greater than twice the one-sided bandwidth), $f_{B}\geq BW.$ In our simulations, we choose $BW\;=\;0.8\;f_{B}$. Let $N_{u}$ be the number of samples in the upbeat, $N_{d}$ be the number of samples in the downbeat, $N_{0}$ be the number of samples during phase 0 and $N_{1}$ be the number of samples during phase 1. Additionally, $N_{u}$ and $N_{d}$ are both integer multiples of 2. The discrete version of the chirp frequency f[k] in the baseband and chirp waveform g[k] are given as follows:

(5) $$f[k]=\left\{ \begin{array}{cc} 2b_{u}T_{B}\cdot \left(\mathrm{mod}\left(\mathrm{mod}\left(k,N_{0}+N_{1}\right),N_{u}+N_{d}\right)-0.5N_{u}\right), & \mathrm{for}\mathrm{the}\mathrm{upbeats}\\ 2b_{d}T_{B}\cdot \left(\mathrm{mod}\left(N_{u}+N_{d}-\mathrm{mod}\left(k,N_{0}+N_{1}\right),N_{u}+N_{d}\right)-0.5N_{d}\right), & \mathrm{for}\mathrm{the}\mathrm{downbeats}\\ -b_{d}T_{B}\cdot N_{d}, & \mathrm{for}\mathrm{the}\mathrm{frequency}\mathrm{hold}\mathrm{periods} \end{array}\right.$$

(6) $$g\left[k\right]=e^{2\pi jT_{B}\sum_{l=0}^{k}f\left[l\right]}$$

where k is the discrete time index.

We assume that the transmitter and the receiver are powered up at the same time. For the radar received signal model, please refer to Section 2.3 of [27]. At the receiver, the FFT processing selects FFT windows centered at $k_{1}=\frac{2}{3}N_{u}+\;n\left(N_{0}+N_{1}\right)$ for the upbeat signal and at $k_{2}=\frac{2}{3}N_{d}+\;N_{u}+n\left(N_{0}+N_{1}\right)$ for the downbeat signal, where integer n represents the $n^{th}$ PRI and starts from 0. Additionally, $N_{u}$ and $N_{d}$ are both integer multiples of 3. This choice of FFT windows can be found in [27].

Based on [18], the radar range resolution has the following lower bound:

(7) $$\Delta R=\frac{c}{2BW}$$

where c is the speed of light. In our design, the FFT window duration is smaller than $T_{u}$ and $T_{d}$. The actual range resolutions for the upbeat and downbeat are denoted by $\Delta R_{upbeat}$ and $\Delta R_{downbeat}$, respectively:

(8) $$\Delta R_{upbeat}=\frac{c\cdot \tau_{1}}{2}=\frac{cf_{B}}{4b_{u}N_{FFT}}$$

(9) $$\Delta R_{downbeat}=\frac{c\cdot \tau_{2}}{2}=\frac{cf_{B}}{4b_{d}N_{FFT}}$$

where $\tau_{1}$ is the time to sweep the frequency range of $\frac{f_{B}}{N_{FFT}}$ in the upbeat sweep, $\tau_{2}$ is the time to sweep the frequency range of $\frac{f_{B}}{N_{FFT}}$ in the downbeat sweep and $N_{FFT}$ is the FFT size. In the simulations, $b_{u}$ and $b_{d}\;$ are equal, therefore, $\Delta R_{upbeat}$ and $\Delta R_{downbeat}$ are equal as well. Therefore, instead of using actual range resolutions for the upbeat and the downbeat, we just mention the actual range resolution for this design in the Results section.

## 3. Digital Phased Array Receiver

In this section, the proposed DPA receiver is elaborated. We describe the receiver architecture in Section 3.1. The per-antenna staggered PRI MTI filter implementation is presented in Section 3.2. The slow time integration module is proposed in Section 3.3. The Doppler estimation module is described in Section 3.4.

### 3.1. Receiver Diagram

**Figure 5:**
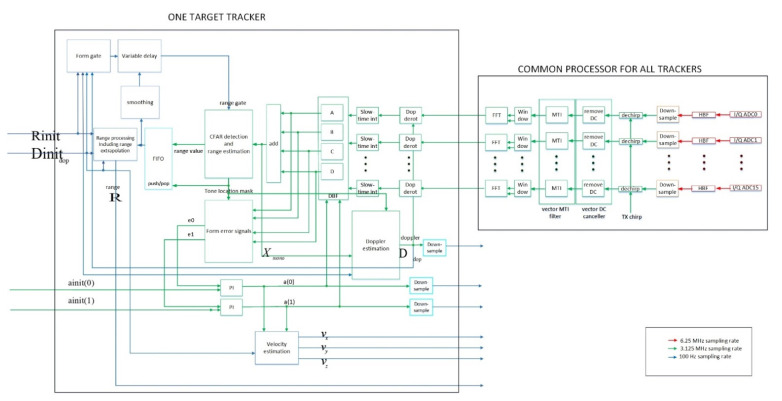
The receiver block diagram. HBF, DBF, PI, FIFO and CFAR stand for half-band filter, digital beamforming, proportional–integral, first-in-first-out and constant false alarm rate, respectively. The quantities $e_{0}$ and $e_{1}$ are monopulse tracking error signals for a(0) and a(1). Please refer to [27] for some of the mathematical details of these blocks.

The proposed receiver uses digital phased array beamforming and multiple target tracking. It is shown in Figure 5. For each target, there is a dedicated tracker. To optimize the hardware resources, the common processor for all trackers is established. Compared with the architecture in [27], this receiver has some differences. First, the common processor for all trackers uses per-antenna FFT. As mentioned in [27], when the number of receive antennas is large, using per-antenna FFT makes hardware implementation complex. In this paper, we focus on the scenario where the number of receive antennas is no more than 32. Second, the per-antenna MTI filter uses the TPS-PRI-FMCW waveform. Third, the slow time integration (the “Slow-time int” block in Figure 5) is introduced after the Doppler de-rotation in the frequency domain. Though Doppler de-rotation can be done in the time domain better than in the frequency domain, it provides time domain processing (done by the common processor) coupled with the frequency domain target tracking operations. Due to this reason, the Doppler de-rotation (the “Dop derot” block in Figure 5) is done in the frequency domain to separate each target tracker from the common processor. Fourth, we introduce a Doppler estimation module. This module takes the signal after the monopulse combining, the tone location of the CFAR detector and the initial normalized Doppler value as inputs, and outputs the Doppler estimate used for Doppler de-rotation.

The Doppler tracking loop (from “Dop derot” to “Slow-time int” to “DBF” to “add” then to “Doppler estimation”) shares the signal $X_{mono}$ with the monopulse angle tracking loop (from “DBF” to “form error signals” then to “PI controllers”), as shown in Figure 5. The DBF module is common to both the Doppler tracking loop and the monopulse angle tracking loop. Therefore, there is a coupling effect between these two loops: the smaller the angle number estimation errors, the smaller the Doppler estimation error and vice versa.

### 3.2. Per-RX Antenna Staggered PRI MTI Filter Implementation

**Figure 6:**
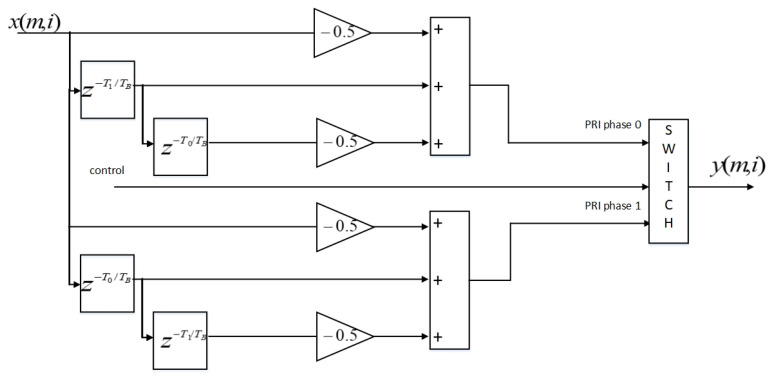
Switched implementation of the staggered pulse repetition interval (PRI) moving target indication (MTI) filter. The numbers m and i are indices of the RX antenna elements along the x and z axes (please refer to Figure 8 of [27]).

The staggered PRI MTI filter is implemented as two parallel MTI filters. The filter coefficients are both [−0.5, 1, −0.5]. The output signals of the two parallel MTI filters are switched by the control signal that indicates the signal phases. This implementation is shown in Figure 6. Bare metal is used for the parameter choice of $T_{0}$, $T_{1}$ and $T_{B}$, where *T_B_* is the duration of a baseband sample and $T_{B}=\frac{1}{f_{B}}$ (*f_B_* is the baseband sampling rate). It is different from the method used in [34] where two variable delay signal buffers are used. The advantage of this implementation is that it can make the operating frequency higher than that when using variable delay signal buffers because it only consists of fixed delays, fixed gains, additions and a switch module. The disadvantages are doubling the usage of hardware resources and no flexibility in changing the delays after implementation.

### 3.3. Slow Time Signal Integration Module

In theory, FFT is a parallel operation with a vector input and a vector output. For real-time DSP processing, the input to the FFT processing is first de-serialized into a vector. Then FFT processing is performed in a vector format. Lastly, the output frequency domain signal of FFT is serialized. This is shown in Figure 5.

The signal $X_{m,i,fft}$ denotes the signal right after FFT for the (*m*,*i*)*th* RX antenna element. The TPS-PRI-FMCW waveform is periodic in $T=T_{0}+T_{1}$. The output signal of the FFT processing retains the periodicity of the input time domain signal. The slow time integration module leverages the periodicity property of the transmitted signal to coherently add the signals at every $f_{B}\cdot T$ samples (the number of samples in one PRI) after the FFT processing. The slow time integration module is simply denoted as:

(10) $$X_{m,i,slti}\left[p,n\right]=\;\frac{1}{K}\overset{K-1}{\underset{q=0}{\sum }}X_{m,i,fft}\left[p,n-q\right]$$

where n is the nth T period, K is the number of coherently added signals and p is the index of the post-FFT signal within the nth period, where $p\;=\;0,1\ldots ,\;f_{B}T$.

If the residual normalized Doppler frequency after the Doppler estimation and de-rotation is zero, the number of coherently added signals can be large. However, the initial normalized Doppler estimates for the tracking radar are not perfect, where the estimation error is denoted by $e_{D}$. Note that the normalized Doppler error, which is unitless, can be translated to the Doppler error (*e_d_*) in Hz by $e_{d}=\;e_{D}f_{B}$.

On the other hand, both $T_{0}$ and $T_{1}$ contain two FFT windows. Additionally, the FFT size determines the FFT processing gain. Therefore, the number of coherently added signals K in fact should satisfy $K<\frac{0.25}{f_{B}\cdot T\cdot e_{D}}+1$ to avoid signal power cancellation. This simply means that $\left|2\pi \left(K-1\right)f_{B}Te_{D}\right|<\frac{\pi }{2}$, where $2\pi \left(K-1\right)f_{B}Te_{D}$ denotes the maximum signal phase rotation introduced by the residual Doppler error over a time interval of $\left(K-1\right)f_{B}T$ and |·| stands for the absolute value. To avoid signal power cancellation, we need to keep the maximum signal phase rotation within

$$\frac{\pi }{2}$$.

In Section 4, K is chosen to be 3 and this gives 4.77 dB of extra processing gain.

### 3.4. Doppler Estimation Module

**Figure 7:**
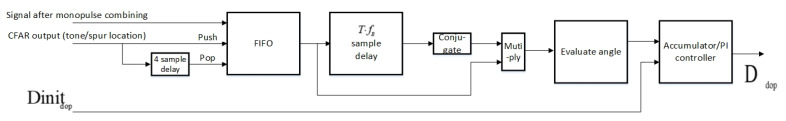
The Doppler estimation module.

The Doppler estimation module uses the signal after monopulse combining $X_{mono}\left[p,n\right]$, the CFAR detector output and the initial normalized Doppler parameter as the inputs. The block diagram is shown in Figure 7. The instantaneous Doppler estimation value can be estimated as:

(11) $$\bar{D_{dop}\left[n\right]}=\frac{∠\left(X_{mono}\left[I_{tone},n\right]\cdot X_{mono}{\left[I_{tone},n-1\right]}^{*}\right)}{2\pi f_{B}T}$$

where ${\left(\cdot \right)}^{*}$ stands for conjugate operation, $I_{tone}$ is the tone index corresponding to where the CFAR detector output is Boolean value true and ∠(·) denotes evaluating the angle (in radians) of the quantity in the brackets. Equation (11) simply calculates the differential phase of $X_{mono}$ at the tone location between the *n*th PRI and the (n−1)th PRI, then normalizes the value by *T*, *f_B_* and 2*π*. The accumulator after evaluating $\bar{D_{dop}\left[n\right]}$ is the classical proportional–integral (PI) controller (please refer to Figure 17 of [27] for an implementation of a proportional–integral (PI) controller), which takes the instantaneous Doppler estimation value $\bar{D_{dop}\left[n\right]}$ and the initial Doppler parameter ${Dinit}_{dop}$ as the inputs and outputs the estimated Doppler value *D_dop_*[*n*].

The monopulse tracking of the two-dimensional angles has tracking errors. These errors also propagate in the Doppler tracking loop. This error propagation effect also affects the slow time integration performance and reduces the maximum number of coherently added signals in the slow time signal integration module.

## 4. Results and Discussion

In this section, we describe the FPGA-in-the-loop simulation setup, the simulation parameters, the simulation results and some discussion about the simulation results. The FPGA-in-the-loop simulation setup is a faster signal processing test bed than the pure computer-based simulation.

### 4.1. The FPGA-in-the-Loop Simulation Test Bed

**Figure 8:**
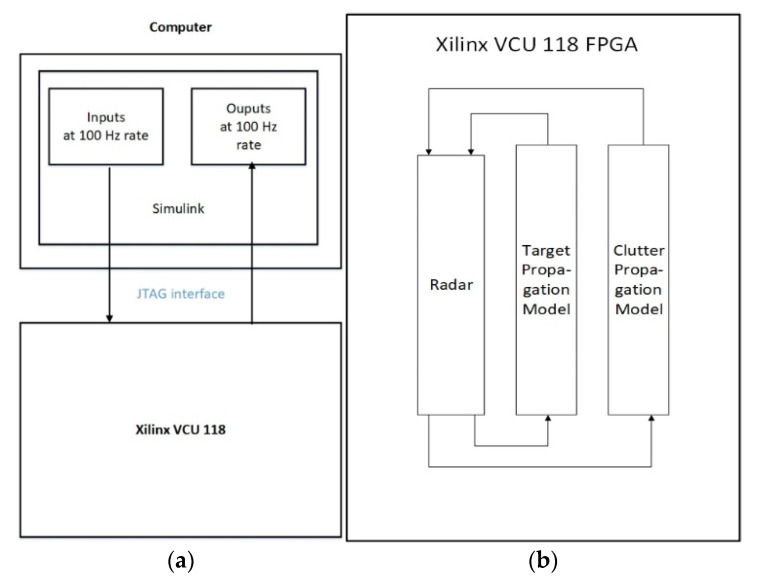
(**a**) The field programmable gate array (FPGA)-in-the-loop block diagram. JTAG stands for the joint test action group. (**b**) the zoom-in plot of the Xilinx VCU 118 FPGA block in (**a**), where a high-level block diagram of the implementation is shown.

The tracking radar system and the proposed algorithm are implemented in MathWorks’ Simulink software. All the modules are carefully modeled in a 32-bit fixed point format. With the FPGA-in-the-loop capability of Simulink, this implementation is programmed on a Xilinx FPGA board (Xilinx Virtex Ultrascale + VCU 118 FPGA) and is interfaced with the computer that runs Simulink. Without the FPGA board, Simulink can simulate the radar transmitter, the line-of-sight RF propagation and the radar receiver in both floating points and fixed points. However, the simulation speed is usually slow. With the FPGA-in-the-loop configuration, as shown in Figure 8, the simulation speed can be improved significantly.

The target propagation and clutter propagation models are simulated at $f_{s}=\;2f_{B}$. The TX waveform is generated in the discrete time of sampling rate $f_{B}$ according to Equations (5) and (6). The signal is upsampled by a factor of 2 to the oversampling rate domain of sampling rate $f_{s}$. Then the signal is filtered by the TX half-band filter to reject the frequency domain signal image caused by the upsampling operation. The radar receiver uses the RX half-band filtering as an anti-aliasing filter and downsampling by a factor of 2, as illustrated in Figure 5, as the initial receiver signal processing operations.

Given the FPGA resource limitation, we only implemented one target tracker for the multi-target tracking platform. The RF propagation module simulates a moving target and a stationary clutter that is close to the initial location of the moving target. The simulation parameter setup will be discussed in Section 4.2.

The inputs to the FPGA simulation are the target parameters, e.g., initial coordinates, initial velocities and accelerations for target modeling generated by the System Tool Kit (STK) software and the initial radar parameters as described in Section 2.2. The constants, like the carrier frequency, the speed of light, the radar PRI, etc., are directly used by the FPGA and are not considered as inputs. The outputs of the FPGA simulation are true values of the target, i.e., the target range, target velocities, target Doppler and target angels, and the radar estimates, i.e., the target range estimates, target velocity estimates, target Doppler estimates and target angle estimates. The inputs and outputs are all updated every 0.01 s of real time.

With STK, a target movement trajectory is created. The target velocities in the radar reference coordinates, i.e., the x, y and z axes, and target coordinates in the x, y and z axes over time and the target RCS over time, are generated by STK. The time granularity of these data sets is 0.01 s. These data sets are fed to Simulink, then Simulink passes the data to the FPGA via the joint test action group (JTAG) interface at a 100 Hz rate. The simulations of radar signal processing and line-of-sight RF propagation are done on the FPGA. The outputs of the FPGA contain the true values and the radar estimated values of the two-dimensional angle numbers, the target range, the normalized Doppler value and the target velocities in the radar reference coordinates. The outputs of the FPGA are also sampled at a rate of 100 Hz, then they are passed to Simulink and are displayed on the display scopes of Simulink. With the FPGA-in-the-loop, we run 16 s of real-time data within 2.5 h of simulation time. This is more than 10 times the speed compared to the pure Simulink-based computer simulation.

The FPGA-in-the-loop implementation uses the hardware description language (HDL) coder design methodology of MathWorks Inc. [43]. We can run the pure computer-based fixed point Simulink model (denoted by run A) and the FPGA-in-the-loop implementation (denoted by run B) concurrently. The outputs of the true values and the estimated values of the angle numbers, the range, the normalized Doppler and the velocities from both runs are compared against each other. We ran 2.5 s of real-time data for run A and run B. The results of run A and run B are identical. Getting the results of run A is computer based. Run A is slow and it is only used for result comparison between run A and run B. With the confidence that the results of run B are identical to run A, we can run longer real-time data for run B. In the later simulation of run B (Section 4.3), we run 16 s of real time data and show simulation results there.

### 4.2. The Simulation Parameters

**Figure 9:**
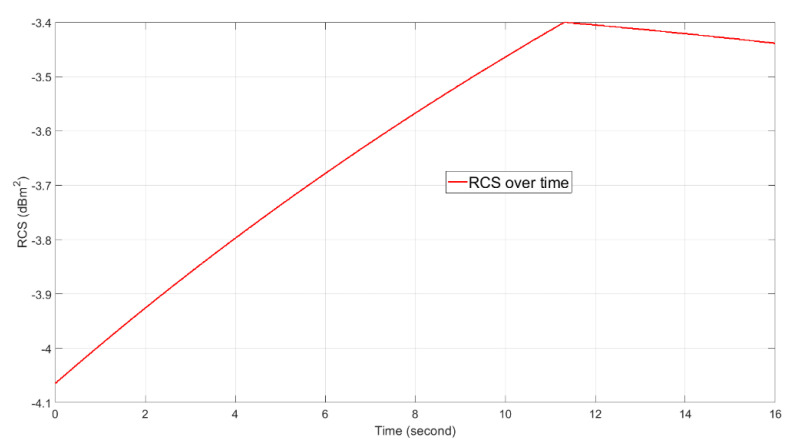
The target RCS vs. time.

We show the simulation parameters in Table 2. Both the TX AESA and the RX DPA use a 4×4 rectangular phased array, as illustrated in Figure 1. Based on the simulation parameters, the actual range resolution of the radar is 113.9 m using Equations (8) and (9). In addition, we illustrate the target RCS vs. time in Figure 9. Additionally, Chebyshev windows of length 1024 are applied before the FFT operations. The window function gives 60 dB sidelobe suppression and causes an SNR loss of 1.8134 dB.

Given these simulation parameters, we assume the initial Doppler parameter has an absolute error that satisfies $\left|e_{d}\right|<50\;\mathrm{Hz}$ and the absolute error is about 13% of 1/T. We can derive that the number of coherently added signals in the slow time signal integration module should approximately satisfy K< 2.91. We choose because K = 3 gives more gain than K = 2 at a 50 Hz initial Doppler error.

### 4.3. Simulation Results

**Figure 10:**
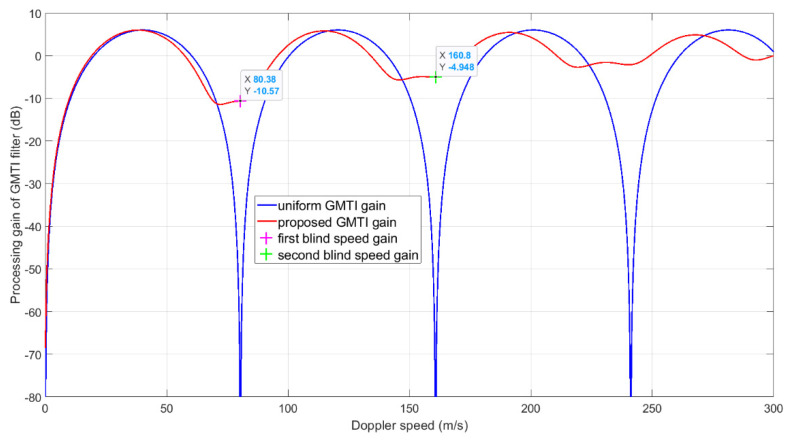
The MTI filter gain vs. Doppler speed for the given simulation parameters. We show the absolute value of the Doppler velocity in the graph. Essentially, the negative Doppler velocity part is symmetrical to the positive Doppler velocity part.

We first show the staggered PRI MTI filter gain vs. Doppler speed in Figure 10. With the uniform PRI MTI filter, the SNR drops near any integer multiples of the blind speed (the blind speed is about 80.4 m/s) are significant. The first null of the uniform PRI MTI filter nulls the clutter signal. This property is retained by the proposed TPS-PRI-MTI design. The nulls of the proposed design at other blind speed points are significantly reduced. Near the second blind speed point (twice the blind speed), the loss is 4.948 dB. Our simulation scenario happens in the second blind speed zone. The velocities in the x, y and z axes are −183, −113 and −18 m/s, respectively, corresponding to −151.5 m/s Doppler speed at time 0. The target moves into the second blind speed zone at −160.8 m/s at a later time.

**Figure 11:**
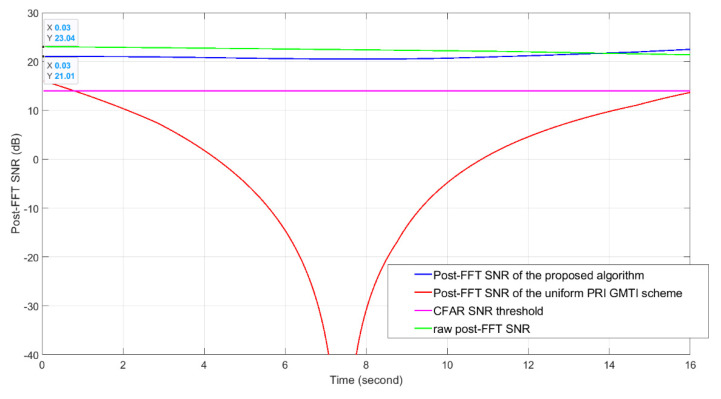
The post-FFT signal to noise ratios (SNRs) vs. time. These data sets are calculated based on the 4×4 AESA mainlobe gain of 12.04 dBi and 4×4 DPA mainlobe gain of 12.04 dBi.

We illustrate the post-FFT SNR vs. time in Figure 11. The green curve shows the raw post-FFT SNR ($SNR_{raw}$) defined in Equation (12).

(12) $$\begin{array}{c} SNR_{raw}=P_{t,Total}+G_{AESA}+G_{DPA}+\;\sigma_{t}+10\cdot \log_{10}\left(\frac{\lambda^{2}}{{\left(4\pi \right)}^{3}R_{tx}^{2}R_{rx}^{2}}\;\right)-noise_{fl}\\ -10\cdot \log_{10}\left(f_{B}\right)+G_{FFT}-L_{Chebywin}. \end{array}$$

This calculation of the raw post-FFT SNR uses the radar range equation based on the range and RCS, per-element TX antenna gain and RX antenna gain, the AESA total power ($P_{t,Total}$) of 79.04 dBm ($P_{t,Total}=\;P_{t}+10\cdot \log_{10}\left(N_{tx}M_{tx}\right)$), the AESA antenna gain ($G_{AESA}$) of 18.04 dBi ($G_{AESA}=\;G_{tR,t}\;+10\cdot \log_{10}\left(N_{tx}M_{tx}\right))$, the DPA gain ($G_{DPA}$) of 18.04 dBi ($G_{DPA}=\;G_{rR,t}\;+10\cdot \log_{10}\left(N_{rx}M_{rx}\right))$, FFT processing gain ($\;G_{FFT}$) of 30.1 dB and the Chebyshev window SNR loss ($L_{Chebywin}$) of 1.81 dB. The quantity $\sigma_{t}$ stands for the target RCS in dB.

The blue curve shows the post-FFT SNR of the proposed algorithm. It considers the summation of the raw post-FFT SNR, the MTI filter noise enhancement of 1.76 dB, the proposed staggered PRI MTI filter gain shown in Figure 10 and the slow time integration gain of 4.77 dB. The red curve shows the post-FFT SNR of the uniform PRI MTI filter. It considers the summation of the raw post-FFT SNR, the MTI filter noise enhancement of 1.76 dB, the uniform PRI MTI filter gain and the slow time integration gain of 7.78 dB (since in this case, the PRI of uniform PRI MTI scheme (using only one-phase PRI instead of two) is about half of the PRI of the proposed scheme). Clearly, the proposed algorithm gives a much higher post-FFT SNR than the uniform PRI scheme. The post-FFT SNR of the proposed algorithm is about 2 dB lower than the raw post-FFT SNR at time 0.03 s. In Figure 11, near 14 s, the SNR of the proposed algorithm even outperforms the raw post-FFT SNR due to the changes of the proposed MTI filter gain over time. Note that in this simulation setup, the target range increases over time because the target moves away from the radar. This causes an SNR decrease over time and offsets the RCS increase over time.

**Figure 12:**
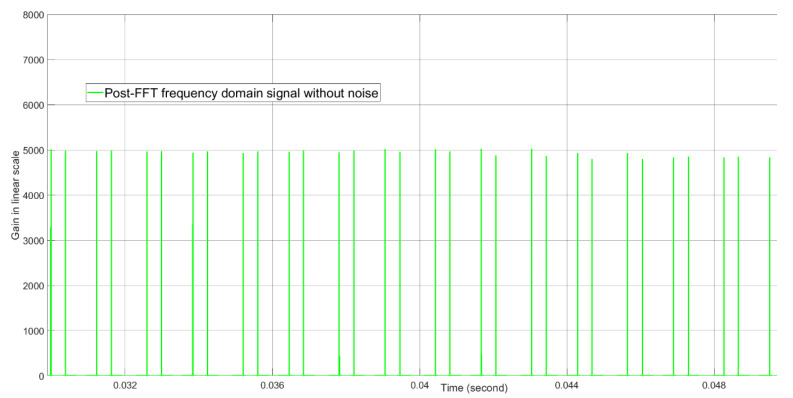
The post-FFT desired tone energy without noise when initial Doppler is equal to the true Doppler at about 0.04 s real time. This result is generated by computer simulation, not by FPGA simulation.

**Figure 13:**
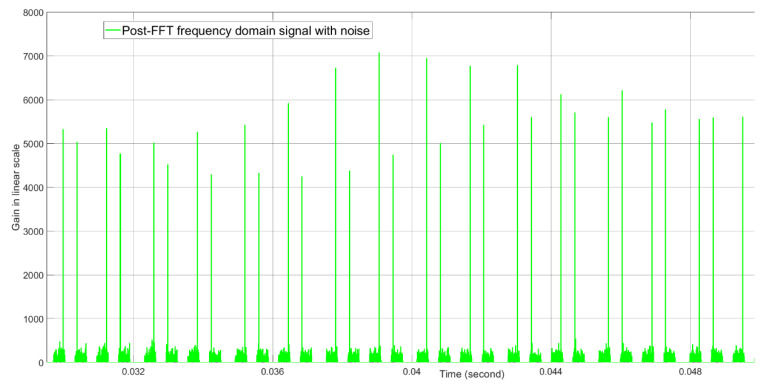
The post-FFT desired tone energy with noise when initial Doppler is equal to the true Doppler at about 0.04 s real time. This result is generated by computer simulation, not by FPGA simulation.

The post-FFT desired tone energies without noise and with noise when the initial Doppler parameter for the tracking radar is equal to the true Doppler value are shown in Figure 12 and Figure 13, respectively. Without noise, the tone energy of the target is about 5000. The calculated tone energy based on the 12.04 dBi of both the AESA antenna gain and the DPA antenna gain is about 5649.8. The difference is 0.53 dB and is mainly because the actual AESA and DPA beamforming gain are both 0.25 dBi lower than 12.04 dBi when the beamforming angles in azimuth and elevation are (1.3476,1.5096) radians instead of $\left(\frac{\pi }{2},\frac{\pi }{2}\right)$ radians. From Figure 12 and Figure 13, with noise, the tone energy shows a much bigger fluctuation than without noise.

**Figure 14:**
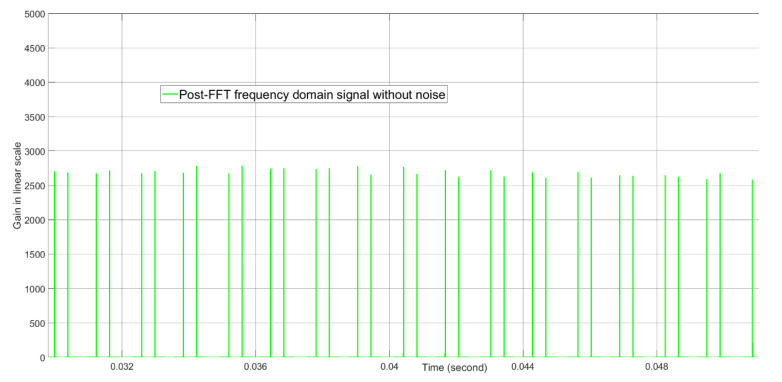
The post-FFT desired tone energy without noise when the initial Doppler estimate has an error of $-1.783\times 10^{-5}\left(-55.7\;\mathrm{Hz}\right)$ at about 0.04 s real time. This result is generated by computer simulation, not by FPGA simulation.

**Figure 15:**
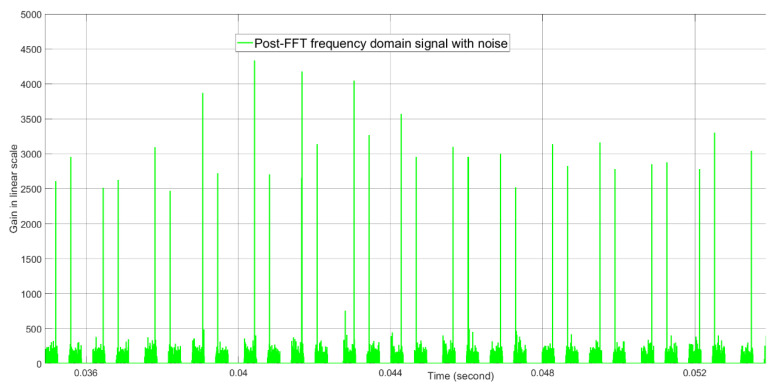
The post-FFT desired tone energy with noise when the initial Doppler estimate has an error of $-1.783\times 10^{-5}\left(-55.7\;\mathrm{Hz}\right)$ at about 0.04 s real time. This result is generated by computer simulation, not by FPGA simulation.

We also illustrate the effects of a non-ideal initial Doppler parameter (as an initial input to the tracking radar) in Figure 14 and Figure 15. Without noise, the tone energy of the target with 55.7 Hz of the Doppler estimation error is almost 2.7 dB lower than the tone energy of the target with no Doppler estimation error. This roughly matches the de-coherence loss of 2.6 dB directly computed from the slow time integration with the Doppler estimation error.

**Figure 16:**
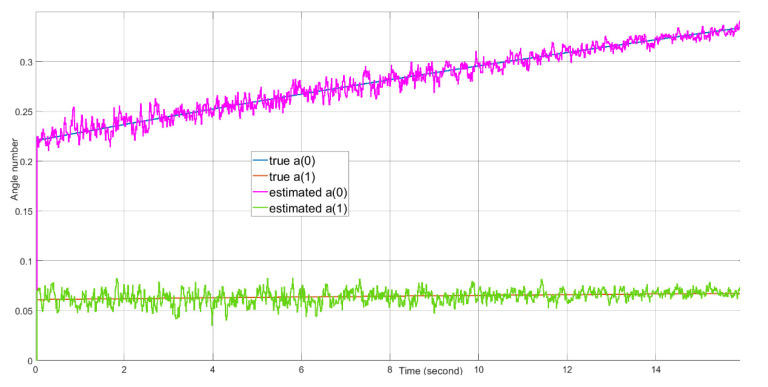
The tracking performance of angle numbers a(0) and a(1) over time.

**Figure 17:**
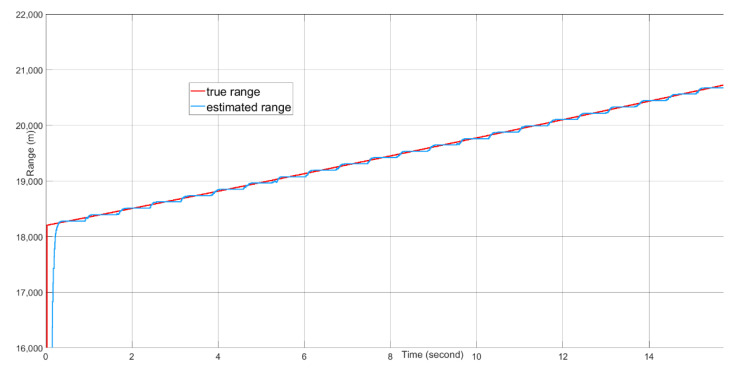
The range tracking performance over time.

**Figure 18:**
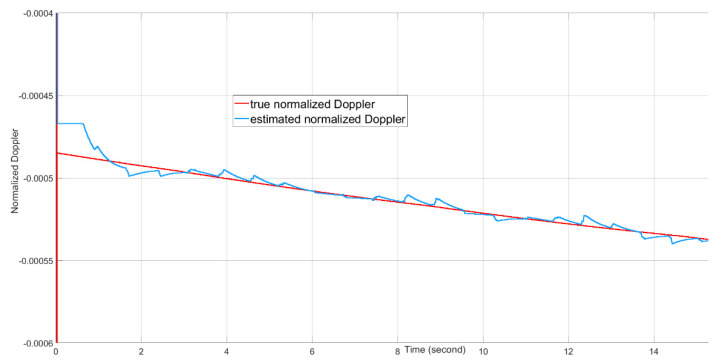
The normalized Doppler tracking performance over time. The estimation error ramps up from time to time. This is likely due to the interaction of the Doppler tracking loop and the monopulse angle tracking loop, as shown in Figure 5.

**Figure 19:**
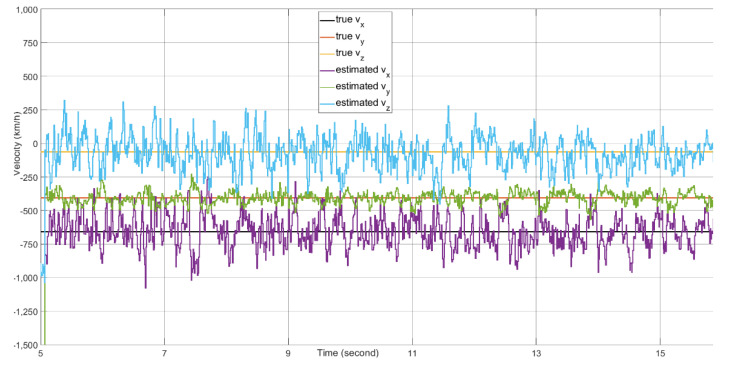
The velocity tracking performance over time. The velocity estimation formula follows Equation (37) in [27]. The estimation interval is 5 s. Therefore, this plot starts from 5 s.

Target tracking of 16 s of real-time data with the presence of a 20 dBm^2^ stationary clutter in the proximity of the target is simulated on the FPGA-in-the-loop test bed. Because the 4×4 AESA and 4×4 DPA produce wide beamwidths, the difference between the beamforming gains of the clutter skin return and the target skin return is no more than 3 dB. The target signal suppression is mostly from the MTI filter. The angle tracking results are plotted in Figure 16. The range tracking results are shown in Figure 17. The Doppler tracking results are shown in Figure 18. The velocity tracking results are shown in Figure 19. At about 20 dB post-FFT SNR, as shown in Figure 11, the tracking estimates of the target angle numbers, the target range and the target Doppler value all match well with the true values of the target angle numbers, the target range and the target Doppler. There are Doppler error ramping-ups (about $5\times 10^{-6}$ in error magnitude) during the target tracking. This is likely due to the coupling of the Doppler tracking loop and the monopulse angle tracking loop, as shown in Figure 5.

One note is that the FPGA initializes all internal states and all the outputs to zeros at time 0, when the FPGA is powered up. We cannot force the initial values of the FPGA to take any other values. It takes 0.02 s of real time to initialize the FPGA internal states to the desired initial values since the time granularity for parameter updates is 0.01 s. After the internal states are initialized at 0.02 s, the true values of the target are outputted. However, it still takes time to update the radar estimates from all zeros due to signal processing delay. For example, the range estimation uses the order 0 IIR filter smoothing operated at a 100 Hz sampling rate. The filter has the form of $\frac{0.25}{1-0.75z^{-1}}$. After 0.02 s, it takes additional transient time (more than 0.04 s) to drive the range estimate from zero to the stable value. For the Doppler tracking, the tracking happens after the range tracking converges. We freeze the Doppler estimate to the initial Doppler value ${\mathrm{Dinit}}_{dop}$ in the first 0.6 s of real time. When we calculate the tracking errors, we ignore the transient behavior, and only focus on the stable outputs.

The measured root mean square tracking errors are shown in Table 3. The angle tracking errors are less than 0.004 radians, corresponding to about 0.2 degrees. The range tracing error is about 28 m. The closed loop Doppler tracking gives a small residual RMS error of 6.6 Hz. The tracking errors are mainly caused by the AWGN noise. For the angle and Doppler tracking, the residual tracking errors caused by the non-ideal responses of the tracking filters or PI controllers are negligible compared to the errors caused by the AWGN noise in this parameter setup. For the range tracking, we observe that the maximal range tracking error is about half of the actual range resolution. The range tracking error is mainly caused by the actual range resolution. The velocity errors are derived from both the angle tracking errors and the range tracking error.

### 4.4. Discussion

This paper proposes a two-phase staggered PRI triangular FMCW signal and the bare-metal staggered PRI MTI filter to address the radar blind speed problem. This solution is more effective than using a uniform PRI FMCW waveform and relying on the range extrapolation solution in [27] because there is a major post-FFT SNR improvement of the proposed solution in the blind speed zone compared with the uniform PRI waveform. The combination of the staggered PRI solution and the range extrapolation can be useful for the scenarios where the staggered PRI cannot remedy the SNR loss in the blind speed zones, for example, in the first blind speed zone.

The introduction of a Doppler tracking loop makes slow time integration possible. However, it has more stringent requirements for the initial Doppler estimation than not employing the Doppler tracking loop because the initial Doppler estimation error reduces the post-processing SNR (the initial Doppler estimation can be done by search radars). In the worst case of high Doppler estimation error, the target tracking process cannot be started.

The multi-target tracking aspect is covered by this paper. Multi-target tracking with AESA is possible only when the multiple targets share the same TX beam or when simultaneous multi-beam TX beamforming is supported. The multi-beam TX beamforming reduces the EIRP of each beam. A careful study of the AESA beam scheduling can be found in [44,45] and is outside the scope of our paper.

The digital signal processing part of the radar design is reported in this paper. We are working on the RF system and the antenna system using mainly off-the-shelf devices. The next step is to integrate the FPGA design into the RF and antenna systems and design and implement the calibration algorithms for the whole system.

## 5. Conclusions

A simple yet effective two-phase staggered PRI FMCW signal and the corresponding receiver MTI filter are proposed for the first time in this paper. The TPS-PRI scheme has a smaller period than the staggered PRI schemes with more than two phases. This enables a new design that has slow time signal integration and a Doppler tracking loop in the frequency domain to boost the post-FFT SNR. The combined TPS triangular FMCW solution and the slow time signal integration algorithm gives only a 2 dB SNR loss compared to the raw post-FFT SNR. We deploy this combined algorithm on the FPGA-in-the-loop radar test bed. This test bed achieves more than 10 times the simulation speed compared with the pure computer-based simulation. The simulation results of 16 s of real-time radar tracking show that the RMS angle tracking error, the RMS range tracking error and the RMS Doppler tracking error are about 0.2 degrees, 28 m and 6.6 Hz, respectively, at 20 dB post-FFT SNR.

## Figures and Tables

**Table 1:** Notations of the TPS triangular FMCW waveform.

Notation	Definition
$f_{c}$	The carrier frequency. No frequency hopping assumed.
$T_{u}$	The upbeat time duration.
$T_{d}$	The downbeat time duration.
$T_{0}$	The phase 0 duration of the TPS triangular FMCW waveform.
$T_{1}$	The phase 1 duration of the TPS triangular FMCW waveform.
$b_{u}$	The constant upbeat chirp rate.
$b_{d}$	The constant downbeat chirp rate.

**Table 2:** The simulation parameters.

Variable Name.	Value	Definition
$M_{tx}$	4	Number of TX antenna elements on the x axis.
$N_{tx}$	4	Number of TX antenna elements on the z axis.
$M_{rx}$	4	Number of RX antenna elements on the x axis.
$N_{rx}$	4	Number of RX antenna elements on the z axis
$d_{TR}$	3 m	The distance between the transmitter and the receiver phase reference centers.
$h_{0}^{c}$	exp(2πj·rand)	Phases of the channel of the target skin return,$\;j=\sqrt{-1}$ and rand is the uniform random number generator.
aa0	1.3476 radians	The initial azimuth angle of the moving target.
taa0	1.3439 radians	The initial value of the azimuth tracking angle.
ae0	1.5096 radians	The initial elevation angle of the moving target.
tae0	1.5010 radians	The initial value of the elevation tracking angle.
$R_{rx,0}$	18,205.2 m	The initial distance between the radar receiver and the target.
Rinit	18,255.2 m	The initial range parameter for the tracking radar initialization
ainit(0)	cos(taa0)·sin(tae0)	The initial a(0) for the tracking radar initialization
ainit(1)	cos(tae0)	The initial a(1) for the tracking radar initialization
${\mathrm{Dinit}}_{dop}$	variable	The initial Doppler parameter for the tracking radar initialization
*σ*_0_	$-4.065\;{\mathrm{dBm}}^{2}$	Initial RCS of the target.
$h_{1}^{c}$	exp(2πj·rand)	Phases of the channel of the clutter skin return.
aa1	$\frac{\pi }{2}$	The azimuth angle of the clutter.
ae1	$\frac{\pi }{2}$	The elevation angle of the clutter.
$R_{\mathrm{rx},1}$	17,705.2 m	The distance between the radar receiver and the clutter.
$\sigma_{1}$	$20\;{\mathrm{dBm}}^{2}$	RCS of the clutter.
$f_{c}$	1.5 GHz	The carrier frequency of the radar.
$f_{B}$	3.125 MHz	The baseband sampling rate.
BW	2.5 MHz	The FMCW radar bandwidth.
$f_{s}$	$2f_{B}$	Oversampling rate.
v	[−183,−113,−18] m/s	Initial target velocities towards the radar on x, y, z axes when the velocities are positive. Negative velocities show that the target moves away from the radar.
*accel*	[0,0,0,] *m*/*s*^2^	Velocity acceleration of the target towards the radar on x, y, z axes.
T	$2.6112\times 10^{-3}\;s$	The waveform period.
$T_{0}$	$1.24608\times 10^{-3}\;s$	The phase 0 duration.
	$1.36512\times 10^{-3}\;s$	The phase 1 duration.
$T_{u}$	$6.2208\times 10^{-4}\;s$	The upbeat duration.
$T_{d}$	$6.2208\times 10^{-4}\;s$	The downbeat duration.
$b_{u}$	$2.0094\times 10^{9}\;\mathrm{Hz}/s$	The upbeat chirp rate.
$b_{d}$	$2.0094\times 10^{9}\;\mathrm{Hz}/s$	The downbeat chirp rate.
$N_{0}$	3894	The number of samples in phase 0 at $f_{B}$ sampling rate.
$N_{1}$	4266	The number of samples in phase 1 at $f_{B}$ sampling rate.
$N_{u}$	1944	The number of samples in the upbeat at $f_{B}$ sampling rate.
$N_{d}$	1944	The number of samples in the downbeat at $f_{B}$ sampling rate.
$G_{tR,t}$	6 dBi	The TX antenna gain for the target of each TX antenna element.
$G_{tR,c}$	6 dBi	The TX antenna gain for the clutter of each TX antenna element.
$G_{rR,t}$	6 dBi	The RX antenna gain for the target of each RX antenna element.
$G_{rR,c}$	6 dBi	The RX antenna gain for the clutter of each RX antenna element.
$d_{c}/\lambda$	0.5	The ratio of antenna spacing and wavelength.
$P_{t}$	67 dBm	The transmit power of each TX antenna element.
$noise_{fl}$	−166 dBm/Hz	The noise floor at 290 kelvin temperature and including the noise figure of 8 dB.
$HBFord_{tx}$	64	Half-band filter order after TX up-sampling and before each DAC at the transmitter.
$HBFord_{rx}$	32	Half-band filter order before RX down-sampling and after each ADC at the receiver.
lagord	8	Lagrange filter order in the oversampling domain.
lpwr	−25.04 dBm	TX to RX leakage power between each TX antenna and each RX antenna. We assume that the isolation between each TX antenna and each RX antenna is 92.04 dB.
$N_{FFT}$	1024	Number of FFT points.
$I_{avg0}$	0	Range IIR smoothing filter order, the filter response looks like $\;\frac{0.25}{1-0.75z^{-1}}$, where z-transform is with respect to the sampling rate of 100 Hz.
$c_{D,0}$	0.005	The Doppler estimation module accumulator/PI controller gain 0.
$c_{D,1}$	$\frac{1}{256}$	The Doppler estimation module accumulator/PI controller gain 1.
K	3	The number of coherently added signals in the slow time integration module.
$N_{grid}$	256	The number of uniform quantization steps for the [−1,1] range for the AESA angle number quantization grid.

**Table 3:** Measured RMS tracking errors.

Item	Value
a(0)	$3.8\times 10^{-3}\;\mathrm{radians}$
a(1)	$3.6\times 10^{-3}\;\mathrm{radians}$
R (range)	28.06 m
$D_{dop}$ (the normalized Doppler value)	$2.1\times 10^{-6}$ (6.6 Hz)

## Data Availability

All simulated data except the Simulink model and the FPGA code are available to readers.
